# Different somatostatin and CXCR4 chemokine receptor expression in gastroenteropancreatic neuroendocrine neoplasms depending on their origin

**DOI:** 10.1038/s41598-019-39607-2

**Published:** 2019-03-13

**Authors:** Rebekka Mai, Daniel Kaemmerer, Tina Träger, Elisa Neubauer, Jörg Sänger, Richard P. Baum, Stefan Schulz, Amelie Lupp

**Affiliations:** 1 Institute of Pharmacology and Toxicology, Jena University Hospital, Jena, Germany; 2 Department of General and Visceral Surgery, Zentralklinik Bad Berka, Bad Berka, Germany; 3 Institute of Pathology and Cytology Bad Berka, Bad Berka, Germany; 4 Center for Molecular Radiotherapy and Molecular Imaging, Zentralklinik Bad Berka, Bad Berka, Germany

## Abstract

Somatostatin receptors (SST), especially SST2A, are known for their overexpression in well-differentiated gastroenteropancreatic neuroendocrine neoplasms (GEP-NEN). The chemokine receptor CXCR4, in contrast, is considered to be present mainly in highly proliferative and advanced tumors. However, comprehensive data are still lacking on potential differences in SST or CXCR4 expression pattern in GEP-NEN in dependence on the place of origin. Overall, 412 samples from 165 GEP-NEN patients, comprising both primary tumors (PT) and metastases (MTS), originating from different parts of the gastrointestinal tract or the pancreas were evaluated for SST and CXCR4 expression by means of immunohistochemistry using monoclonal antibodies. SST2A was present in 85% of PT with a high intensity of expression, followed by SST5 (23%), CXCR4 (21%), SST3 (10%), SST1 (9%), and SST4 (4%). PT displayed higher SST2A and chromogranin A (CgA) expression levels than MTS. In both PT and MTS lower SST2A and CgA expression levels were found in tumors originating from the appendix or colon, compared to tumors from other origins. Tumors derived from appendix or colon were associated with significantly worse patient outcomes. Positive correlations were noted between SST2A and CgA as well as between CXCR4 and Ki-67 expression levels. SST2A and CgA negativity of the tumors was significantly associated with poor patient outcomes. All in all, SST2A was the most prominent receptor expressed in the GEP-NEN samples investigated. However, expression levels varied considerably depending on the location of the primary tumor.

## Introduction

Somatostatin receptors (SST), especially SST2A, are well known for their overexpression in well-differentiated gastroenteropancreatic neuroendocrine neoplasms (GEP-NEN), where they serve as the molecular basis for SST-based diagnostics and treatment modalities.

The chemokine receptor CXCR4, in contrast, is considered to be present mainly in highly proliferative and advanced tumors. In many studies, it has been demonstrated, that elevated CXCR4 expression is associated with rapid tumor progression, high invasiveness, early metastasis, and poor patient outcome^1,2^. Recently, it has been shown that SST2A expression gradually declines with increasing malignancy from G1 neuroendocrine tumors to G3 neuroendocrine carcinomas^3–13^, whereas CXCR4 expression is increased^14–16^. Correspondingly, in GEP-NEN SST2A positivity of the tumor has been associated with better patient outcome^3,6,8–10,12,17^, whereas presence of CXCR4 has been related to low overall survival^15^.

Apart from that, it has been suggested that malignancy as well as overall survival rates may differ depending on the localization of the GEP-NEN along the gastrointestinal tract, with higher malignancy (Ki-67 levels) and thus lower survival rates in hindgut as compared to foregut tumors^18^. Since such distinctions have implications for diagnostic and therapeutic procedures, it would be of interest to know if there are also differences in SST or CXCR4 expression patterns in GEP-NEN of different anatomical origin. Due to the scarcity of GEP-NEN cases comprehensive data on this issue are still lacking. Therefore, the aim of the present study was to re-evaluate SST and CXCR4 expression in a large set of formalin-fixed, paraffin-embedded GEP-NEN samples originating from stomach, duodenum/jejunum, ileum, appendix, colon, rectum or pancreas by using well characterized rabbit monoclonal antibodies^19–23^ and to correlate the expression with clinical data.

## Methods

### Tumor specimens

A total of 412 archived formalin-fixed, paraffin-embedded tumor samples from 165 patients (in detail, 61 × 1, 41 × 2, 30 × 3, 20 × 4, 7 × 5, 2 × 6, 2 × 7, 1 × 10, and 2 × 14 samples per patient) with histologically verified gastroenteropancreatic neuroendocrine neoplasm (132 primary tumors, 95 metastases) were included in the present investigation. These samples are derived from a different cohort of patients as compared to our previous investigation^7^. Of the tumors, 19 (12%) originated from the stomach, 15 (9%) from the duodenum/jejunum, 59 (36%) from the ileum, 5 (3%) from the appendix, 9 (5%) from the colon, 14 (8%) from the rectum, and 39 (24%) from the pancreas. From 5 tumors (3%) localization of the primary was unknown. The samples were provided by the Institute of Pathology and Cytology Bad Berka, Bad Berka, Germany, and had been surgically removed between 1999 and 2014 at the Department of General and Visceral Surgery, Zentralklinik Bad Berka, Bad Berka, Germany. The clinical data were gathered from the patient records. In 82 of the 165 patients an SST-based PET/CT had been performed. The PET/CT scans were processed with Siemens e.soft Nuclear Medicine Workstation. With the help of this software, automatic region of interest was drawn on the individual tumor lesions and SUVmax values were calculated. To avoid major influence of partial volume effect on lesion SUVmax, region of interest was drawn only on lesions greater than 1.5 cm in size.

Permission was gained from the local ethics committee (Ethikkommission der Landesärztekammer Thüringen) for this retrospective analysis. All data were recorded and analyzed anonymously.

### Immunohistochemistry

**Figure 1:**
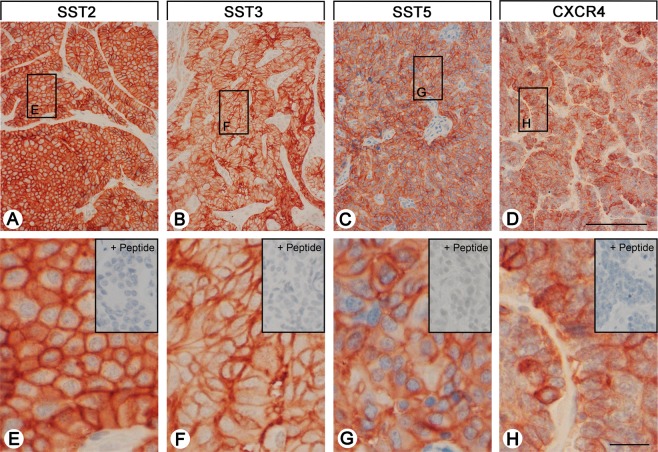
SST and CXCR4 expression pattern in gastroenteropancreatic neuroendocrine neoplasms (GEP-NEN). Depicted are typical examples of staining patterns for SST2A, SST3, SST5, and CXCR4. Immunohistochemistry (red-brown color), counterstaining with hematoxylin; scale bar: 500 µm (**A**–**D**), 50 µm (**E**–**H**). Insets in **E**–**H**: for adsorption controls the anti-SST antibodies and the anti-CXCR4 antibody were incubated with 10 µg/ml of the peptide used for immunizations (+Peptide).

From the paraffin blocks, 4 µm sections were prepared and floated onto positively charged slides. Immunostaining was performed by an indirect peroxidase labeling method as described previously^24^. Rabbit monoclonal antibodies (hybridoma cell culture supernatants) directed against the respective carboxyl-terminal tails of the receptors were used to detect SSTs (except for SST4) and CXCR4 (for detailed information on the clones, epitopes, and the dilutions of the antibodies, see Supplementary Table 5). With respect to SST4, similar, but polyclonal, antibodies were applied. Sections from human pancreas (islets; SST1, SST2A, SST3, SST5), lymph nodes (germinal centers; SST2A, SST5, CXCR4), and human cortex (SST4) served as positive controls. As negative control, the primary antibody was either omitted or adsorbed for 2 h at room temperature with 10 µg/ml of the peptide used for immunizations. In all cases a complete abolition of immunostaining was observed (see insets in Fig. 1 and in Supplementary Fig. 1). Additional stainings were performed with monoclonal mouse antibodies against the proliferation marker Ki-67 and against chromogranin A (CgA), a marker for neuroendocrine tumors (Supplementary Table 5).

Staining for the receptors as well as for CgA in all sections was scored by means of the semiquantitative Immunoreactivity Score (IRS) according to Remmele and Stegner^25^. The percentage of positive tumor cells quantified in five gradations (no positive cells (0), <10% positive cells (1), 10–50% positive cells (2), 51–80% positive cells (3), >80% positive cells (4)) was multiplied by the staining intensity quantified in four gradations (no staining (0), mild staining (1), moderate staining (2), strong staining (3)). Thus, IRS values ranging from 0 to 12 were obtained. Tumor samples with average IRS values ≥3 were considered positive. With the antibodies against the SSTs and the CXCR4 distinct immunostaining of the plasma membrane, but also of the cytoplasm of the tumor cells was seen, which reflects receptor internalization due to agonist stimulation. Both types of staining, cytoplasmatic and cell surface staining, were evaluated equally. With respect to Ki-67 staining, the percentage of positive nuclei was determined. All immunohistochemical stainings were evaluated by two independent blinded investigators (RM, AL). In case of discrepant scores, final decision was achieved by consensus.

### Statistics

For statistical analysis, the IBM SPSS statistics program version 22.0.0.0 was used. Because the data were not normally distributed (Kolmogorov-Smirnov test), Kruskal-Wallis test, Mann-Whitney test, Chi-Square test, Kendall’s τ-b test, and Spearman’s rank correlation were performed. For survival analysis, the Kaplan-Meier method with a log-rank test was used. P values ≤ 0.05 were considered statistically significant. In cases where one patient had more than one tumor slide, an arithmetic mean was calculated from the IRS values of all the slides of this patient, primary tumor and metastasis/es taken together (per patient analysis). Only when primary tumors and metastases were compared, arithmetic means were calculated for the primary tumor samples and metastasis/es sample(s) separately.

### Ethics approval and consent to participate

All procedures performed in this study involving human participants were in accordance with the 1964 Helsinki declaration and its later amendments. Permission was gained from the local ethics committee (Ethikkommission der Landesärztekammer Thüringen) for this retrospective analysis. Informed consent for the use of tissue samples for scientific purposes was obtained from all individual participants included in the study when entering the Theranostic Research Center, Zentralklinik Bad Berka, Bad Berka, Germany. All data were analyzed anonymously.

## Results

### Patient characteristics

In total, tumors from 94 male (57% of the cases) and 71 female patients (43%) were evaluated in the present investigation. Mean age of the patients at diagnosis was 58.8 years overall (median: 59.0 years, range: 12.1–85.0 years), with some differences in dependence on the localization of the primary tumor (Kruskal-Wallis test: p = 0.082; Supplementary Table 1). Patients having tumors originating from the colon were diagnosed at a significantly older age as compared to those with tumors from the stomach, duodenum/jejunum, ileum or pancreas (pairwise Mann-Whitney tests: p < 0.050). Twenty-three of the (corresponding) primary tumors (14%) were classified as T1, 22 (13%) as T2, 51 (31%) as T3, and 21 (13%) as T4. In 48 cases (29%), the extent of the primary tumor was unknown. Thirty-six of the patients (22%) had no lymph node metastases, whereas in 101 cases (61%) lymph node metastases were already present. For 28 tumors (17%) lymph node status was not known. Forty-nine patients (30%) had no distant metastases, in 97 patients (59%) distant metastases were already present, and in 19 cases (11%) the existence of distant metastases was unknown (Supplementary Table 2). Distant metastases were mainly found in the liver, but were present also in other parts of the gastrointestinal tract or the pancreas, in the peritoneum, retroperitoneum, omentum, mesenterium, mesocolon, brain, thyroid, lung, gallbladder, lig. hepatoduodenale, spleen, adrenals, fat capsule of the kidney, kidney, ovary, testis, abdominal adipose tissue, abdominal wall, bones or skin. At diagnosis, 17 patients (10%) had UICC stage I disease, 8 patients (6%) had stage II disease, 23 patients (14%) had stage III disease, and 97 patients (58%) had already stage IV disease. From 20 patients (12%) the stage of the disease was unknown (Supplementary Table 3). With respect to histological grading, 80 patients (48.5%) had grade 1, 61 patients (37%) had grade 2, and 24 patients (14.5%) displayed grade 3 histology (Supplementary Table 3). Regarding functionality, 101 tumors (61.2%) were non-functional and 62 (37.6%) functional. In 2 cases (1.2%) functionality of the tumor was not reported in the patient files. Median survival was 4.0 years overall (minimum: 0 months, maximum: 29.3 years). Here, patients with tumors originating from appendix or colon displayed significantly worse outcomes compared to patients with other derivations of the primary tumor (pairwise log-rank tests: p < 0.05; Supplementary Table 1; Supplementary Fig. 2). Interestingly, this seems to be independent of the presence of lymph node or distant MTS, as e.g. tumors from the ileum already displayed lymph node MTS in 86% (colon: 77.8%), and distant MTS in 73% (colon: 66.7%) of the cases (Supplementary Table 2). There were, however, significant differences between the primary tumor localizations regarding tumor size/infiltrating growth and grading (χ^2^ test: p < 0.001). At diagnosis in stomach, duodenum/jejunum, ileum and rectum pT4 tumors were present in 11.1%, 27.3%, 17.4%, 0% and 12.1% of the cases and G3 tumors in 15.8%, 6.7%, 0%, 21.4% and 20.5% of the patients, respectively. By contrast, in appendix and colon pT4 tumors were diagnosed in already 50% of the patients and G3 tumors in 40% and 55.6% of the cases, respectively (Supplementary Tables 2, 3).

All data of the individual patients including gender, age, localisation of the primary tumor, type and derivation of the samples investigated as well as prior therapies are depicted in the Supplementary Table 6.

### Somatostatin and CXCR4 chemokine receptor expression pattern

Figure 1 and Supplementary Fig. 1 show examples of staining with the monoclonal antibodies against SST1, 2A, 3 and 5, and CXCR4. Distinct immunostaining of the plasma membrane, but also of the cytoplasm of the tumor cells was seen, which may reflect receptor internalization due to agonist stimulation. The polyclonal anti-SST4 antibody, in contrast, showed only cytoplasmic positivity (Supplementary Fig. 1).

**Figure 2:**
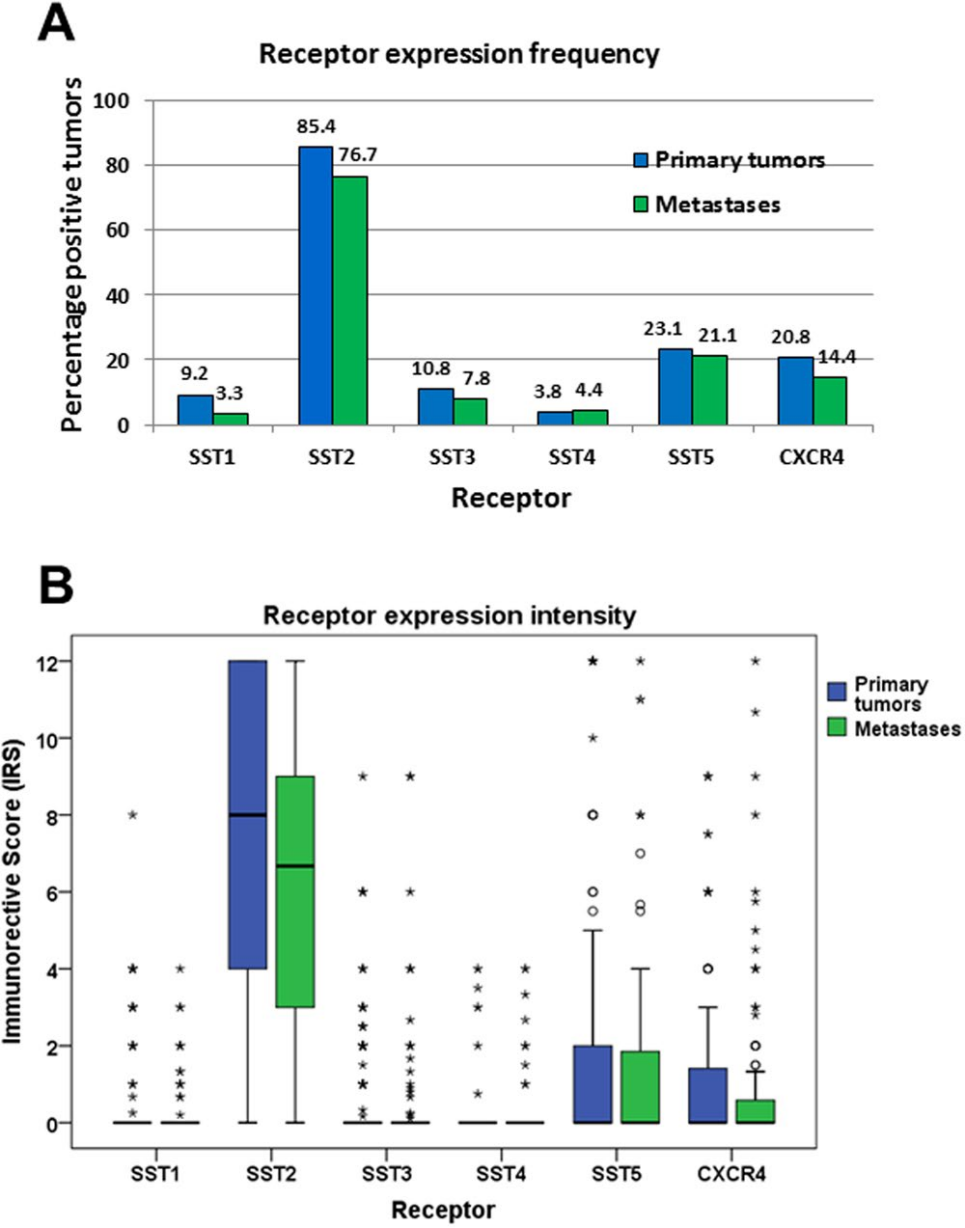
Expression profiles of different somatostatin receptor (SST) subtypes and CXCR4 chemokine receptor in primary tumors and metastases of gastroenteropancreatic neuroendocrine neoplasms. (**A**) Percentage of positive cases for different SSTs and CXCR4. Tumors were only considered positive at IRS values ≥3. (**B**) Box plots of somatostatin receptor (SST) and CXCR4 expression levels (IRS values). Depicted are median values, upper and lower quartiles, minimum and maximum values, and outliers. Outliers are defined as follows: circles: mild outliers; data that fall between 1.5 and 3 times above the upper quartile or below the lower quartile; asterisks: extreme outliers; data that fall more than 3 times above the upper quartile or below the lower quartile.

Overall, SST2A was by far the most prominently expressed receptor in the GEP-NEN samples investigated (Fig. 2A,B), followed by SST5, CXCR4, SST3, SST1 and SST4.

For all receptors, expression levels varied considerably between individual patients and sometimes also between different samples from the same patient. This holds true especially for SST2A, where a huge variation was seen in expression levels between different patients, reaching from 0 IRS points (no expression) to 12 IRS points (maximum expression), which is illustrated by the length of the respective boxes and whiskers in Fig. 2B. To evaluate the extent of intratumor heterogeneity in receptor expression, the mean standard deviation (SD) of the IRS values of the SSTs and of the CXCR4 was calculated from the respective SDs of the individual patients for all samples (primary tumors plus metastases taken together) as well as for the primary tumors (PT) and metastases (MTS) separately. Here, the following results were obtained, showing again the highest variation for the SST2A, but also a similar variation in PT and MTS: Mean SD: SST1: all samples: 1.00, PT: 1.22, MTS: 0.75; SST2A: all samples: 4.21, PT: 4.15, MTS: 4.20; SST3: all samples: 1.47, PT: 1.33, MTS: 1.59; SST4: all samples: 1.11, PT: 0.86, MTS: 1.30; SST5: all samples: 2.81, PT: 2.80, MTS: 2.83; CXCR4: all samples: 2.24, PT: 2.08, MTS: 2.38.

There was a significant positive correlation (p < 0.05) between SST1 levels and SST3, SST4, SST5 and CXCR4 expression, of SST2A levels and SST3 and SST5 expression, of SST3 levels and SST1, SST4 and SST5 expression, of SST4 levels and SST1, SST3, SST5 and CXCR4 expression, and of SST5 levels with those of all other receptors investigated. CXCR4 presence correlated positively with SST1, SST4 and SST5 expression (Table 1).

**Table 1:** Correlations between expression intensities of different somatostatin receptors (SST), CXCR4, chromogranin A (CgA), Ki-67 and the maximum standardized uptake values (SUVmax) in SST-PET/CT scans (data were calculated using mean IRS values of each patient; n = 165).

		SST2A	SST3	SST4	SST5	CXCR4	CgA	Ki-67	SUVmax
**SST1**	**r**	0.054	**0.345**	**0.221**	**0.423**	**0.279**	−0.058	**0.176**	**0.155**
	**p**	0.495	**<0.001**	**0.004**	**<0.001**	**<0.001**	0.461	**0.024**	**0.026**
**SST2A**	**r**		**0.255**	−0.087	**0.156**	0.027	**0.348**	**−0.165**	**0.393**
	**p**		**0.001**	0.268	**0.046**	0.732	**<0.001**	**0.042**	**<0.001**
**SST3**	**r**			**0.180**	**0.291**	0.130	**0.253**	0.052	0.004
	**p**			**0.021**	**<0.001**	0.095	**0.001**	0.505	0.968
**SST4**	**r**				**0.211**	**0.264**	−0.027	0.093	−0.060
	**p**				**0.006**	**0.001**	0.731	0.235	0.594
**SST5**	**r**					**0.237**	−0.078	**0.247**	0.119
	**p**					**<0.001**	0.322	**0.001**	0.089
**CXCR4**	**r**						**−0.173**	**0.262**	0.084
	**p**						**<0.001**	**0.001**	0.455
**CgA**	**r**							**−0.183**	0.003
	**p**							**<0.001**	0.977
**Ki-67**	**r**								0.034
	**p**								0.760

Significant correlations (p < 0.05) are marked in bold; r: correlation coefficient (Spearman); p: p value.

### Somatostatin and CXCR4 receptor expression in dependence on tumor localization

Compared to MTS, PT displayed significantly higher SST2A and Chromogranin A (CgA) expression levels as well as significantly higher maximum standardized uptake values (SUVmax) in the SST-PET/CT (Mann-Whitney test: SST2A: p = 0.032; CgA: p = 0.041; SUVmax: p = 0.020) (Supplementary Fig. 3). No significant differences were observed between PT and MTS with respect to expression levels of the other SSTs and CXCR4 and regarding Ki-67 values (Supplementary Fig. 3). Independently of that, when correlating the IRS values of the SSTs, CXCR4 and CgA, the Ki-67 levels and the SUVmax values of the SST-PET/CT between PT and MTS of the individual patients, significant interrelationships were found for the SST2A (r_sp_ = 0.627; p < 0.001), the SST4 (r_sp_ = 0.371, p = 0.003), the SST5 (r_sp_ = 0.302, p = 0.016), the CXCR4 (r_sp_ = 0.613, p < 0.001), CgA (r_sp_ = 0.405, p = 0.001), the Ki-67 index (r_sp_ = 0.767, p < 0.001) and the SUVmax values of the SST-PET/CTs (r_sp_ = 0.371, p = 0.044).

**Figure 3:**
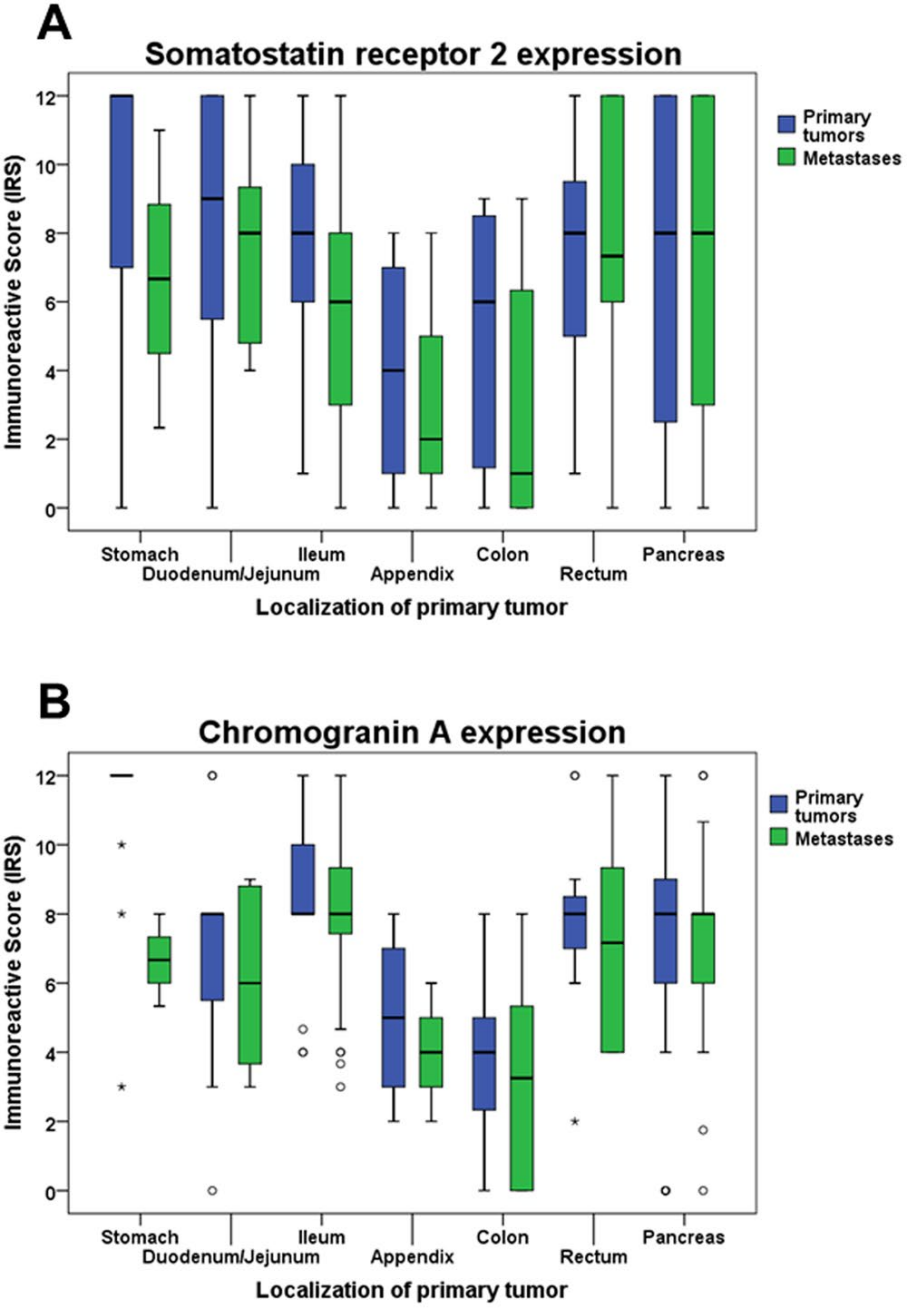
SST2A (**A**) and Chromogranin A (**B**) expression levels in primary tumors and metastases of gastroenteropancreatic neuroendocrine neoplasms of different origin. Depicted are median values, upper and lower quartiles, minimum and maximum values, and outliers. Outliers are defined as follows: circles: mild outliers; data that fall between 1.5 and 3 times above the upper quartile or below the lower quartile; asterisks: extreme outliers; data that fall more than 3 times above the upper quartile or below the lower quartile. Kruskal-Wallis test: (**A**) p = 0.048 (primary tumors); p = 0.055 (metastases); (**B**) p < 0.001 (primary tumors); p = 0.009 (metastases).

**Figure 4:**
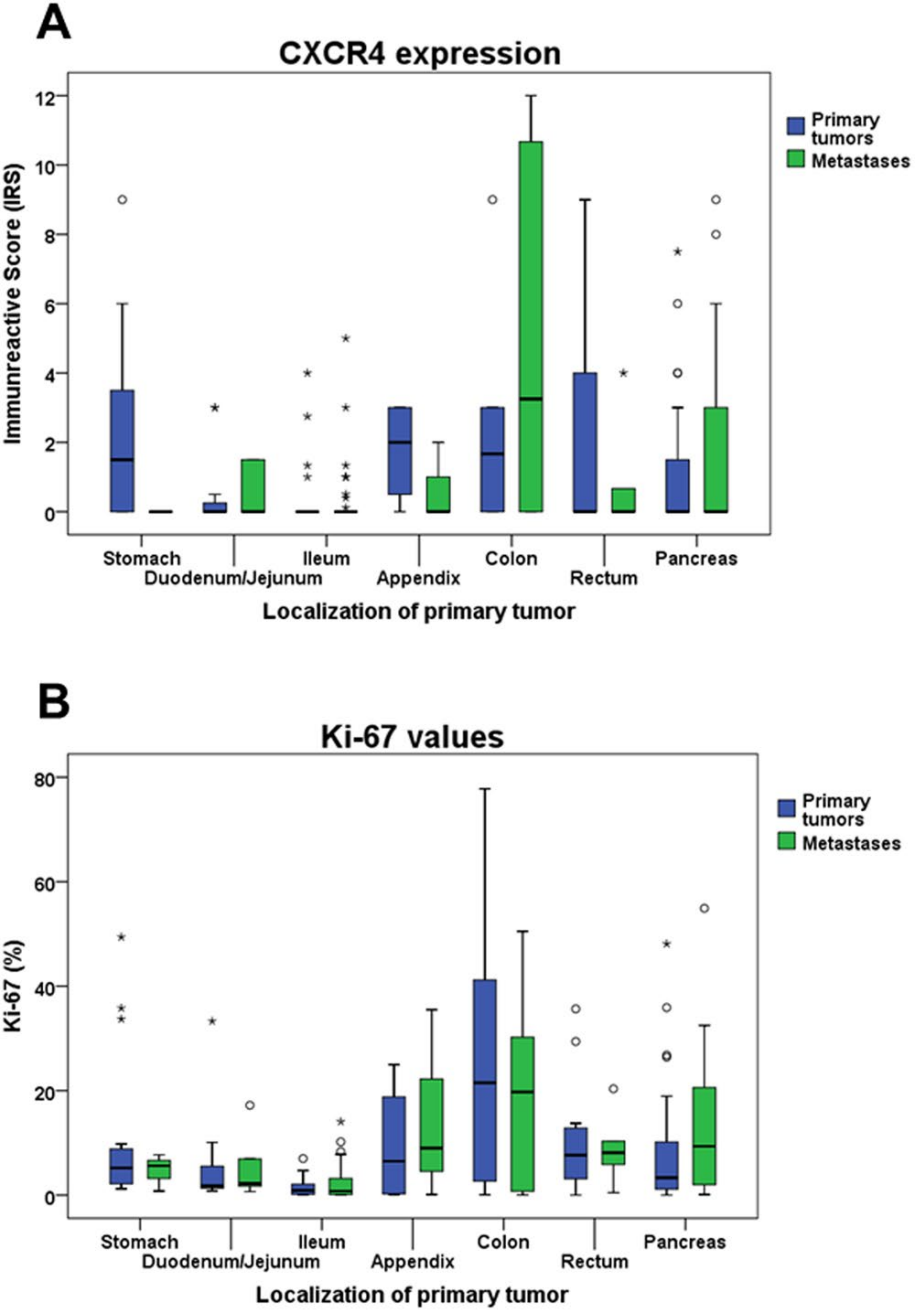
CXCR4 (**A**) and Ki-67 (**B**) expression levels in primary tumors and metastases of gastroenteropancreatic neuroendocrine neoplasms of different origin. Depicted are median values, upper and lower quartiles, minimum and maximum values, and outliers. Outliers are defined as follows: circles: mild outliers; data that fall between 1.5 and 3 times above the upper quartile or below the lower quartile; asterisks: extreme outliers; data that fall more than 3 times above the upper quartile or below the lower quartile. Kruskal-Wallis test: (**A**) p < 0.001 (primary tumors); p = 0.042 (metastases). (**B**) p < 0.001 (primary tumors, metastases).

When comparing tumors from different provenances, dissimilarities were seen with respect to SST2A expression, with significantly lower IRS values in tumors originating from colon or appendix than in those from other origins (Kruskal-Wallis test: p = 0.037; pairwise Mann-Whitney tests: p < 0.05). These differences were also present when considering PT and MTS separately (Kruskal-Wallis test: PT: p = 0.048; MTS: p = 0.055; pairwise Mann-Whitney tests: PT; MTS: p < 0.05 except for appendix/colon vs. rectum or pancreas) (Fig. 3A). Similar results were obtained also for CgA levels (Kruskal-Wallis test: PT plus MTS: p < 0.001; PT: p < 0.001; MTS: p = 0.009; pairwise Mann-Whitney tests: p < 0.05) (Fig. 3B). Significant differences were also seen for CXCR4 expression between the various tumor localizations (Kruskal-Wallis test: PT plus MTS: p = 0.001; PT: p < 0.001; MTS: p = 0.042). However, in this case tumors originating from appendix or colon showed higher CXCR4 expression values than those from other regions (Fig. 4A). In the pairwise comparisons, however, these differences were not statistically significant, except between colon and ileum in PT (Mann-Whitney test: p = 0.024) and MTS samples (Mann-Whitney test: p = 0.020). Differences were also seen with respect to Ki-67 values (Kruskal-Wallis test: PT plus MTS; PT; MTS: p < 0.001). Also here, tumor samples from appendix and colon tended to higher values compared to those from other provenances. However, in the pairwise comparisons significant differences were only detected between colon and ileum samples (Mann-Whitney test: PT plus MTS: p = 0.002; PT: p = 0.009; MTS: p = 0.038) (Fig. 4B).

With all other parameters investigated no relevant differences between the different tumor entities were seen.

### Correlations with clinical data

#### Chromogranin A and Ki-67 expression

There was a significant positive correlation between CgA and SST2A and SST3 expression intensities (Table 1) and also between CgA positivity and SST2A positivity (IRS ≥ 3) of the tumors (χ^2^ test: p < 0.001). Between CgA and CXCR4 expression intensities, in contrast, a negative association was noted (Table 1).

Between the Ki-67 index and SST1, SST5 and CXCR4 expression levels positive correlations were observed, but negative correlations between the Ki-67 index and the SST2A and CgA score values (Table 1).

#### SST-based PET/CT

In 82 of the 165 patients (i.e. 50% of the cases) an SST-based Ga-68 PET/CT had been performed preoperativly; in 66 cases Ga-68 DOTA-TOC [DOTA-D-Phe^1^-Tyr^3^-octreotide] was used, in 9 cases Ga-68 DOTA-TATE [DOTA-(Tyr^3^)-octreotate], and in 6 cases Ga-68 DOTA-NOC [DOTA-[Nal^3^]-octreotide]. SUVmax values of the SST-PET/CT scans showed a significant association with the SST2A expression intensities of the tumors, but (though less pronounced) also with the IRS values for SST1 and SST5 (Table 1). When considering the DOTA-TOC-PET/CT scans separately, a significant interrelationship was seen for SST2A only (r_s_ = 0.450, p < 0.001).

#### TNM status and tumor staging and grading

SST5 and CXCR4 as well as Ki-67 expression intensities significantly correlated with tumor grading (SST5: τ = 0.178, p = 0.007; CXCR4: τ = 0.256, p < 0.001; Ki-67: τ = 0.894, p < 0.001), whereas a negative correlation was noted between SST2A and CgA expression intensities and tumor grading (SST2A: τ = −0.281, p < 0.001; CgA: τ = −0.313, p < 0.005), or tumor size (SST2A: τ = −0.204, p = 0.005; CgA: τ = −0.208, p = 0.005). CgA serum values, in contrast, exhibited a positive association with tumor stage and size (tumor stage: τ = 0.351, p < 0.001; tumor size: τ = 0.280, p = 0.005).

Presence or absence of lymph node or distant MTS had no influence on SST, CXCR4 or CgA expression intensities and also no effect on SUVmax values of the PET/CT scans.

#### Chromogranin A serum levels and Karnofsky performance index

No interrelationship was seen between CgA serum values and CgA expression in the tumor samples (r = 0.039, p = 0.744). There was also no correlation between serum CgA levels and SST or CXCR4 expression in the tumors.

In case of distant metastases, patients showed significantly higher serum CgA levels (Mann-Whitney test: p < 0.001), a reduced Karnofsky index (Mann-Whitney test: p = 0.017) and lower overall survival times (Mann-Whitney test: p = 0.001). Correspondingly, between serum CgA levels and the Karnofsky performance index a negative association was observed (r_s_ = −0.325, p = 0.007).

#### Tumor functionality

Regarding functionality of the tumors, significantly higher CXCR4 expression rates and a tendency towards a higher Ki-67 index were seen in non-functional tumors as compared to functional tumors (Mann-Whitney test: CXCR4: p = 0.019; Ki-67: p = 0.103). CgA serum values, in contrast, were significantly lower in patients with non-functional tumors (Mann-Whitney test: p = 0.006). Functionality, however, was significantly more often associated with distant metastases (distant metastases were present in 81% of the cases in functional tumors and in 56% of the cases in non-functional tumors; χ^2^ test: p = 0.002). All other parameters did not significantly differ between functional and non-functional tumors, including SST expression, overall survival time or patient outcome, respectively.

#### Patient overall survival

**Figure 5:**
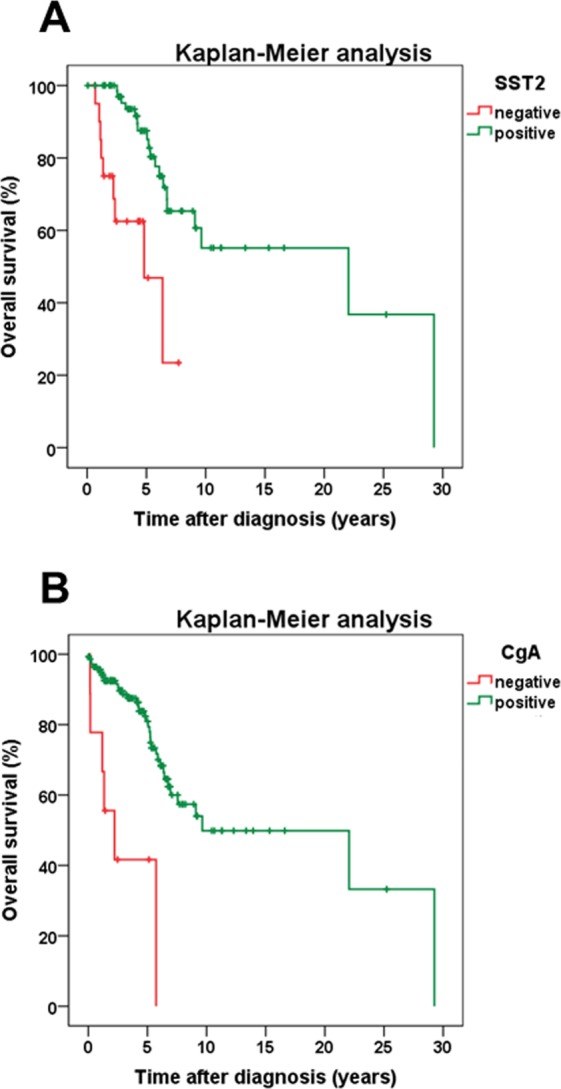
Overall survival of GEP-NEN patients with either no SST2A expression or with SST2A positivity of the tumor (**A**), or with either no chromogranin A (CgA) expression or with CgA positivity of the tumor (**B**). Log-rank test: p < 0.001 (**A**,**B**). Mean survival (years ± SD): patients with SST2A-positive tumors: 5.37 ± 4.73; patients with SST2A-negative tumors: 2.37 ± 1.83; patients with CgA-positive tumors: 4.90 ± 4.51; patients with CgA-negative tumors: 2.08 ± 1.92.

Patient overall survival showed a positive association with SST2A (but not with any other SST) expression intensity (r_s_ = 0.186, p = 0.025) and there was a significantly better outcome of patients with SST2A-positive compared to SST2A-negative tumors (log-rank test: p < 0.001) (Fig. 5A). Similar results were obtained when considering PT and MTS separately (PT; MTS: log-rank test: p < 0.001; Supplementary Fig. 4), when calculating the statistics with ileum-NEN or pancreas-NEN patients alone (ileum; pancreas: log-rank test: p < 0.001; Supplementary Fig. 5) or when considering G1, G2 or G3 tumors, pT3 or pT4 tumors, tumors with or without lymph node MTS (pN0/pN1) or distant MTS (pM0/pM1), IUCC stage 3 or stage 4 tumors, and functional and non-functional tumors separately (in all cases: log-rank test: p < 0.01). Also when SST2A positive cases were grouped according to the median IRS value into cases with moderate (IRS ≤8) or strong (IRS >8) SST2A expression, a significantly better patient outcome was seen for the patients with tumors with strong in comparison to those with neoplasms with moderate SST2A expression (log-rank test: p = 0.005; Supplementary Fig. 6). As with SST2A, significantly better outcomes were also seen in patients with CgA-positive neoplasms compared to CgA-negative tumors (PT plus MTS, PT and MTS separately: log-rank test: p < 0.001; all other cases log-rank test: p < 0.020) (Fig. 5B). On the other hand, patient overall survival time and patient outcome were negatively correlated with Ki-67 levels (r_s_ = −0.286, p < 0.001), patient age (r_s_ = −0.258, p = 0.002), tumor grade (τ = −0.188, p = 0.002; log-rank test: p < 0.001), size (log-rank test: p < 0.001), stage (τ = 0.259, p < 0.001; log-rank test: p < 0.001) and (as mentioned already above) presence of distant MTS. However, multivariate analysis (including IRS values of SSTs, CXCR4 and CgA, Ki-67 levels, patient age, tumor grade, size and stage, tumor functionality, presence of lymph node or distant MTS), revealed SST2A expression and Ki-67 levels as the only independent prognostic factors for patient outcome.

## Discussion

The primary aim of the present study was a comprehensive (re)evaluation of the SST and CXCR4 expression in a large set of GEP-NEN samples of different origin. To the best of our knowledge, the present study represents the largest one investigating all SST subtypes and CXCR4 by immunohistochemistry using well characterized monoclonal antibodies in different GEP-NEN locations including stomach so far. Furthermore, the present study is the largest one investigating SST expression in primary tumors and metastases separately. Additionally, in the present study comprehensive correlations of the SST and CXCR4 expression with a broad range of clinical data were performed, which have never been done so far to this extent and in this detail. To the best of our knowledge there are only two studies available on CXCR4 expression in GEP-NEN so far^15,16^ comprising 64 and 61 cases, respectively; for an overview of the studies of the last 15 years on SST expression in GEP-NEN, including the number of cases evaluated, the SSTs studied, the type of antibodies used and if correlations were performed with tumor functionality, location, grading/staging, Ki-67 index, CgA expression, SST-based imaging or patient outcomes, see Supplementary Table 4.

### Patient characteristics

With a median age at diagnosis of 58.5 years and an almost equal gender distribution, our patient population is very similar to literature data^8,17,18,26–30^. According to previous reports, GEP-NEN patients are often diagnosed at an already advanced stage of the disease^17,18,28,29^. Also in our cohort, 58% of the patients had already stage IV disease at diagnosis, 61% of the cases presented with lymph node MTS, and 59% of the patients had even distant MTS. Most of our tumor samples were originating from the small intestine (in total 45%), followed by pancreas (24%). Similar frequency distributions of GEP-NEN were also reported by other authors^28,29,31^. As observed also in the literature^5,10,12,27–29^, most of the tumors (61.2%) were non-functional, but (as shown also before)^29^ symptoms were more likely with metastatic disease. Median survival of our patients was 4.0 years (48 months), which also corresponds to previous findings^27,29^. Interestingly, and similar to earlier reports^18^. patients having tumors originating from appendix or colon displayed a significantly worse outcome than those having tumors from other provenances and patient outcome appears to be dependent on tumor size/infiltrative growth, metastatic status, stage and grading, as described previously^26^.

### Somatostatin and CXCR4 chemokine receptor expression pattern

In the GEP-NEN samples investigated, SST2A was by far the most significant SST expressed, followed by SST5 and the other SSTs. These results fit well to recent data obtained in GEP-NEN samples and in pancreatic NEN using similar monoclonal antibodies as in the present investigation^8,15^. Whereas strong prevalence of SST2A in GEP-NEN has been shown throughout the literature, regardless of whether polyclonal or monoclonal antibodies have been used, in many studies higher staining results for the other SSTs have been reported^3–5,10,32–36^. In these studies, however, polyclonal antibodies were used, which may have led to an over-estimation of expression rates due to their lower specificity. The same holds true for investigations on CXCR4 expression in GEP-NEN, where higher expression rates were seen with polyclonal antibodies^14,16^ than with monoclonal ones^15^.

### Somatostatin and CXCR4 receptor expression in dependence on tumor localization

In the present study, significantly higher SST2A and CgA expression rates as well as SUVmax PET/CT values were observed in PT as compared to MTS, thus confirming previous results^15^. Additionally and for the first time, differences in receptor expression could be demonstrated depending on the derivation of the tumor sample. Tumors originating from appendix or colon showed lower SST2A and CgA, but higher CXCR4 and Ki-67 expression levels as compared to those from other provenances. Fittingly, patients with tumors derived from appendix or colon showed the worst outcome. These relationships are further substantiated by the fact that in the present study a positive correlation was detected between patient outcome and SST2A or CgA expression, but a negative association with Ki-67 expression.

### Correlations with clinical data

In the present study negative correlations were observed between tumor grading and SST2A and CgA expression, and a positive association with CXCR4 and Ki-67 expression. Similar relationships with tumor grading have been found previously for SST2A expression^3–13^, CXCR4 expression^7,14,16^, and Ki-67 values^3,7,18,31^. Regarding SST5 expression, literature data are still conflicting. While in the present study as well as in another investigation^5^ positive correlations between SST5 expression and tumor grading were found, negative associations have also been reported^4,10,12^. Due to the overall low expression rates of SST5, further studies with a significantly higher number of cases are obviously needed.

The present study revealed a distinct correlation between SST2A and CgA expression, confirming previous results^37^. Therefore, CgA expression could be used as an indirect estimate for SST2A expression levels. Interestingly, there was no interrelationship between tumor CgA expression and serum CgA values. While tumor CgA expression negatively correlates with tumor size, serum CgA values showed a positive interrelationship with tumor size, presence of distant MTS and tumor stage, thus confirming literature data demonstrating an association of serum CgA values with tumor burden and patient outcome^29,30,38–40^.

In the present study also a distinct correlation between SST2A (but also SST1 and SST5) expression and SUVmax values in the PET/CT scans was observed. These results further corroborate numerous data in the literature showing good concordance between SST-based imaging modalities and SST2A (and SST5) expression of the tumors^4,15,17,33,34,36,37,41,42^. High SST2 expression and high uptake in SST-based imaging represent clinically relevant predictors for potential success of subsequent pharmacotherapy with somatostatin analogs or for peptide receptor radionuclide therapy (PRRT)^43–46^.

Regarding the impact of the functionality on malignancy of the tumors and on patient outcome, literature data are controversial^12,47–49^. In the present investigation, no overt difference between functional and non-functional tumors with respect to survival rates and patient outcome could be observed. Additionally, as has been shown previously^5,32^, functionality of the tumors had no influence on SST expression, but there was a significantly lower CgA expression in non-functional as compared to functional tumors. Furthermore, significantly higher CXCR4 and Ki-67 expression levels in non-functional than in functional tumors could be shown, pointing to a somewhat higher malignancy of non-functional tumors.

The present study also shows a clear-cut positive association between SST2A or CgA expression and patient outcome/overall survival. Similar observations have been made by several authors^3,6,8–10,12,17,28^, but (due to the high number of cases) we were able to show this association also for PT and MTS as well as for the different TNM, grading or staging classes or for functional and non-functional tumors separately. We were additionally able to show that not only SST2A positivity, but also the level of SST2A expression is related to prognosis. Similar to the findings in the present study, no impact of any other SST on the course of the disease has been found in the literature^8,9^, with the exception of SST5^10,12^. As expected, Ki-67 levels, tumor size, grade, stage and presence of distant MTS were negatively correlated with patient outcome/overall survival^8,27–29^. Additionally, patient age was confirmed as a negative predictor^27,29^.

## Conclusions

Overall, SST2A was the most prominently expressed receptor in the GEP-NEN samples investigated. Therefore, SST2A-based functional imaging and (if surgery is not possible) SST2A-based therapies (pharmacotherapy with somatostatin analogs, PRRT) should be first choice in metastasized tumor stage. Nevertheless, there was substantial variation in expression levels between individual patients. Expression levels varied considerably depending on the location of the primary tumor, with distinctly lower values in NEN originating from appendix and colon as compared to tumors from other intestinal origins. We also observed significantly lower expression levels in MTS than in PT. SST2A expression declined depending on the malignancy of the tumor and there was a distinct association between SST2A expression and patient outcome.

Since CXCR4 presence was inversely correlated with SST2A expression especially in high-grade neuroendocrine carcinomas of the appendix or colon, this receptor may represent an interesting new target structure, which should be validated in further studies.

## Supplementary information

Supplementary information

## Data Availability

The datasets used and/or analysed during the current study are available from the corresponding author on reasonable request.
