# Supplementary material for: Different somatostatin and CXCR4 chemokine receptor expression in gastroenteropancreatic neuroendocrine neoplasms depending on their origin
Source: Sci Rep. 2019 Mar 13;9:4339. doi: 10.1038/s41598-019-39607-2 (PMC6416272; doi:10.1038/s41598-019-39607-2)
Supplement: Supplementary file 1 — Supplementary information [file 41598_2019_39607_MOESM1_ESM.docx]

**Different somatostatin and CXCR4 chemokine receptor expression in gastroenteropancreatic neuroendocrine neoplasms depending on their origin**

Rebekka Mai, Daniel Kaemmerer, Tina Träger, Elisa Neubauer, Jörg Sänger, Richard P. Baum, Stefan Schulz, Amelie Lupp

**Supplementary Figure 1:**


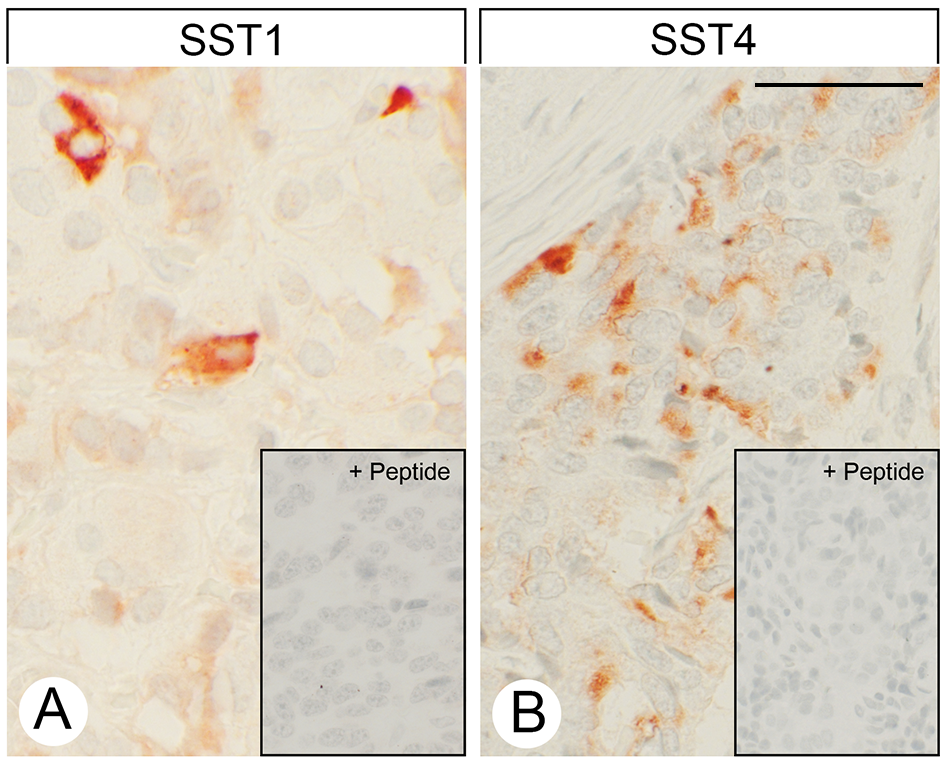


Typical examples of staining patterns for SST1 and SST4 in gastroenteropancreatic neuroendocrine neoplasms (GEP-NEN). Immunohistochemistry (red-brown color), counterstaining with hematoxylin; scale bar: 500 µm. Insets: for adsorption controls the antibodies were incubated with 10 µg/ml of the peptide used for immunizations (+ Peptide).

**Supplementary Figure 2:**


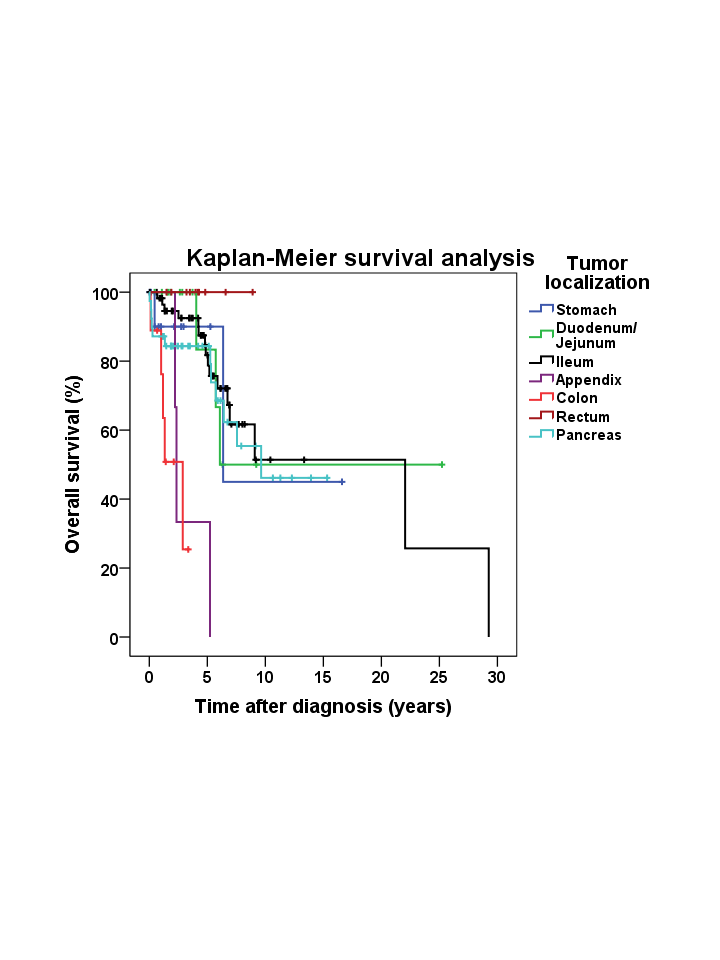


Overall survival of GEP-NEN patients depending on the localization of the primary tumor. Pairwise log-rank-test: appendix, colon vs. other localizations: p < 0.05. Mean survival (years ± SD): stomach: 3.25 ± 5.67; duodenum/jejunum: 5.17 ± 6.27; ileum: 5.45 ± 4.71; appendix: 3.26 ± 1.71; colon: 1.56 ± 1.04; rectum: 4.28 ± 2.17; pancreas: 4.75 ± 4.05; unknown: 5.39 ± 4.01.

**Supplementary Figure 3:**


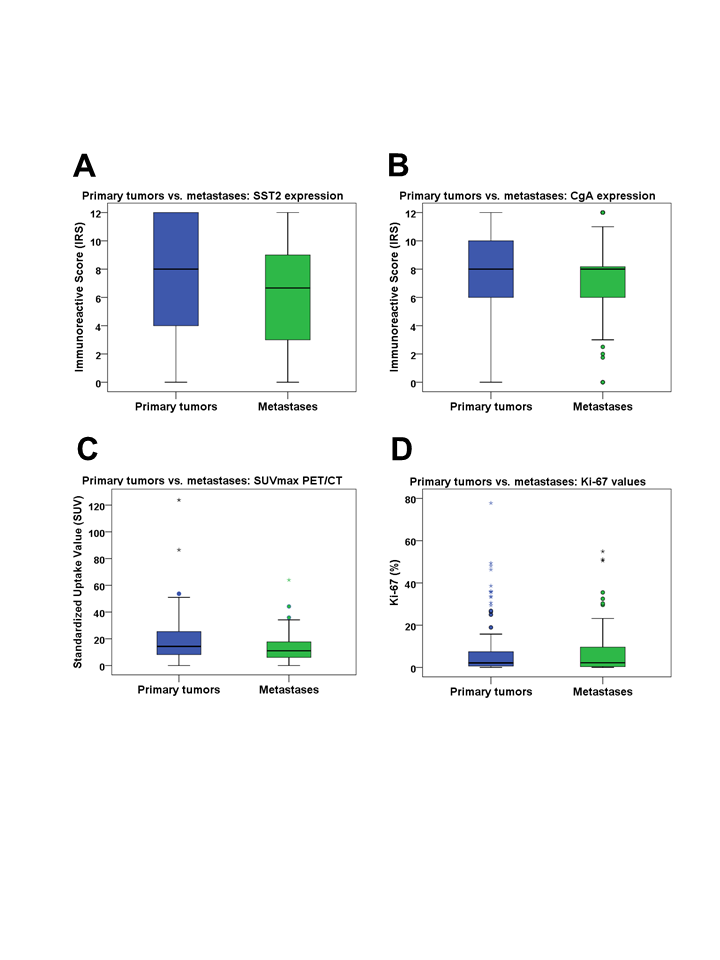


SST2A expression (A), chromogranin A expression (B), maximum standardized uptake values (SUVmax) of SST-based PET/CT scans (C) and Ki-67 levels (D) in primary tumors as compared to metastases. Depicted are median values, upper and lower quartiles, minimum and maximum values, and outliers. Outliers are defined as follows: circles: mild outliers; data that fall between 1.5 and 3 times above the upper quartile or below the lower quartile; asterisks: extreme outliers; data that fall more than 3 times above the upper quartile or below the lower quartile. Mann-Whitney test: (A) p = 0.032; (B) p = 0.041; (C) p = 0.020; (D) p = 0.888.

**Supplementary Figure 4:**


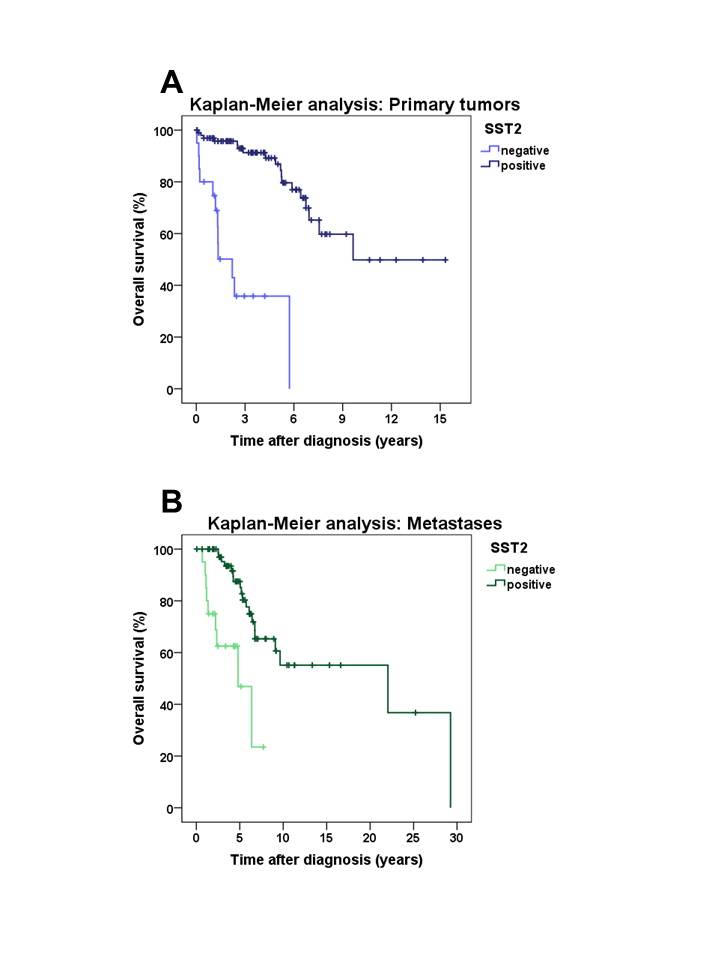


Overall survival of GEP-NEN patients with either no SST2A expression or with SST2A positivity of the tumor. (A) Primary tumors; (B) metastases. Log-rank test: p < 0.001 (A, B). Mean survival (years ± SD): patients with SST2A-positive primary tumors: 4.28 ± 3.08; patients with SST2A-negative primary tumors: 1.72 ± 1.48; patients with SST2A-positive metastases: 6.32 ± 5.17; patients with SST2A-negative metastases: 3.03 ± 2.00.

**Supplementary Figure 5:**


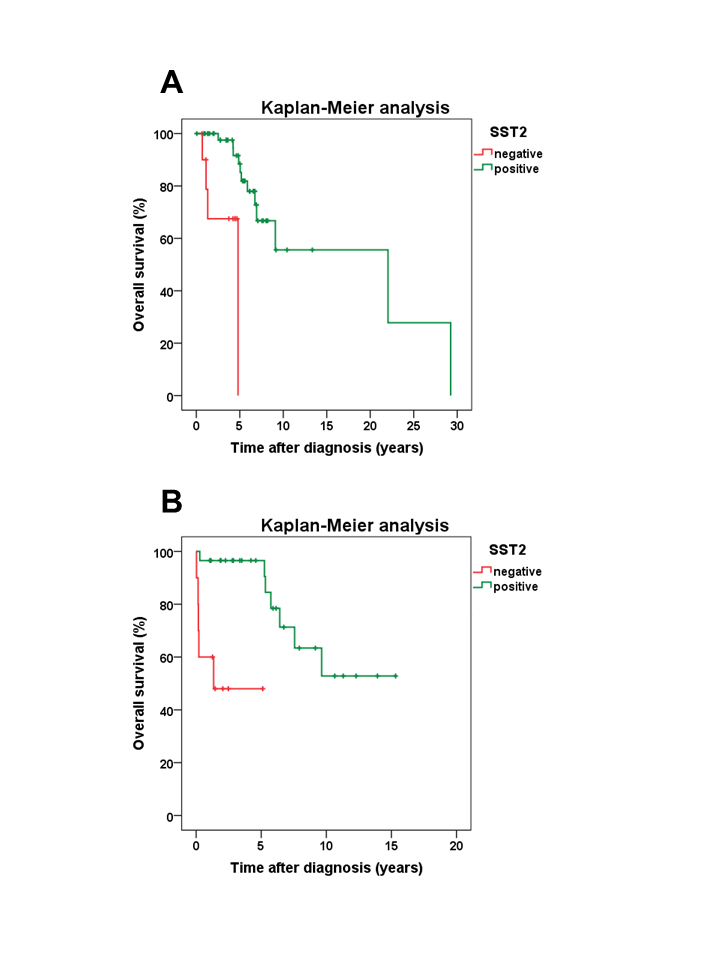


Overall survival of ileum-NEN (A) or pancreas-NEN (B) patients with either no SST2A expression or with SST2A positivity of the tumor. Log-rank test: p < 0.001 (A, B). Mean survival (years ± SD): patients with SST2A-positive ileum-NEN: 6.04 ± 4.97; patients with SST2A-negative ileum-NEN: 2.84 ± 1.81; patients with SST2A-positive pancreas-NEN: 5.89 ± 4.03; patients with SST2A-negative pancreas-NEN: 1.43 ± 1.56.

**Supplementary Figure 6:**


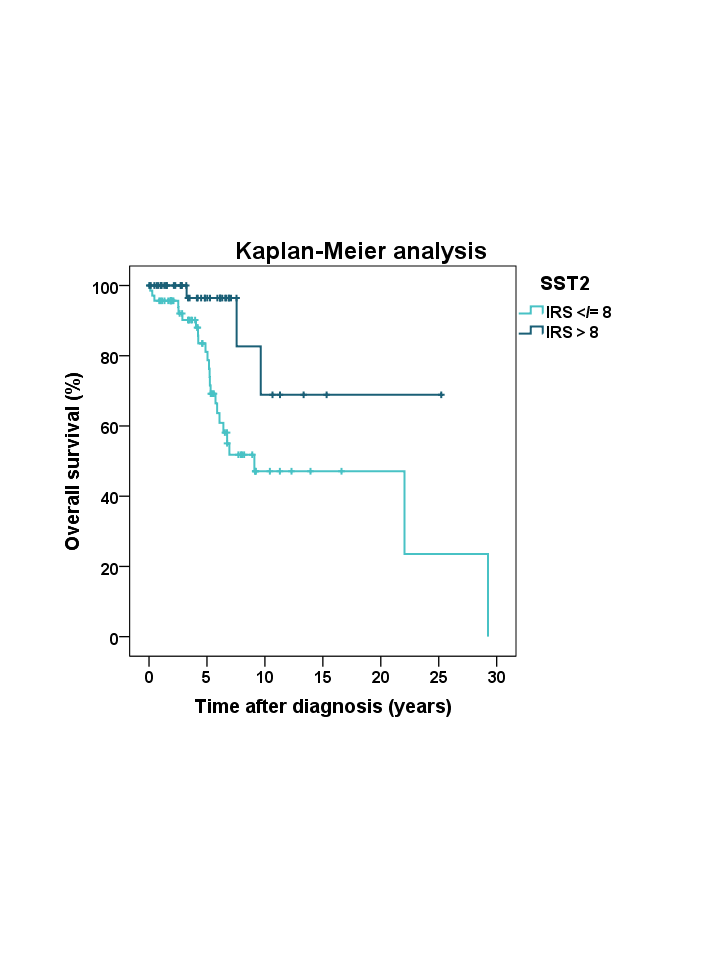


Overall survival of GEP-NEN patients with either moderate SST2A expression (IRS ≤ 8) or with strong (IRS > 8) SST2A positivity of the tumor. Log-rank test: p = 0.005. Mean survival (years ± SD): patients with moderately SST2A-positive tumor: 5.02 ± 4.54; patients with strong SST2A-positive tumor: 5.59 ± 4.83.

**Supplementary Table 1:** Patient characteristics

| **Origin of the primary tumor** | **Cases** | **Gender** | | **Age at diagnosis** | | **Overall survival** | | **Functionality of the tumor** | | |
| --- | --- | --- | --- | --- | --- | --- | --- | --- | --- | --- |
|  | (number) | male  (number) | female  (number) | mean  (years) | median  (years) | mean  (years) | median  (years) | no  (number) | yes  (number) | unknown  (number) |
| **stomach** | 19 | 7 | 12 | 57.9 | 58.3 | 3.25 | 1.58 | 14 | 5 | 0 |
| **duodenum / jejunum** | 15 | 10 | 5 | 57.1 | 59.7 | 5.17 | 3.28 | 7 | 8 | 0 |
| **ileum** | 59 | 32 | 27 | 60.8 | 59.1 | 5.45 | 4.82 | 30 | 29 | 0 |
| **appendix** | 5 | 4 | 1 | 54.0 | 52.3 | 3.26 | 2.34 | 3 | 0 | 2 |
| **colon** | 9 | 7 | 2 | 68.2 | 68.9 | 1.56 | 1.33 | 4 | 5 | 0 |
| **rectum** | 14 | 9 | 5 | 62.3 | 64.1 | 4.28 | 4.07 | 13 | 1 | 0 |
| **pancreas** | 39 | 21 | 18 | 54.2 | 55.4 | 4.75 | 3.50 | 26 | 13 | 0 |
| **unknown** | 5 | 4 | 1 | 55.3 | 58.9 | 5.39 | 3.87 | 4 | 1 | 0 |
| **Σ** | 165 | 94 | 71 | 58.8 | 59.0 | 4.71 | 4.02 | 101 | 62 | 2 |

**Supplementary Table 2:** Tumor characteristics I (unkn.: unknown).

| **Origin of the primary tumor** | **pT**  (number of cases) | | | | | **pN**  (number of cases) | | | **pM**  (number of cases) | | |
| --- | --- | --- | --- | --- | --- | --- | --- | --- | --- | --- | --- |
|  | **1** | **2** | **3** | **4** | **unkn.** | **0** | **1** | **unkn.** | **0** | **1** | **unkn.** |
| **stomach** | 7 | 0 | 1 | 1 | 10 | 5 | 5 | 9 | 6 | 5 | 8 |
| **duodenum / jejunum** | 3 | 3 | 2 | 3 | 4 | 5 | 9 | 1 | 7 | 7 | 1 |
| **ileum** | 2 | 12 | 24 | 8 | 13 | 5 | 51 | 3 | 14 | 43 | 2 |
| **appendix** | 1 | 0 | 0 | 1 | 3 | 2 | 1 | 2 | 2 | 1 | 2 |
| **colon** | 1 | 0 | 3 | 4 | 1 | 1 | 7 | 1 | 3 | 6 | 0 |
| **rectum** | 4 | 0 | 4 | 0 | 6 | 4 | 5 | 5 | 3 | 6 | 5 |
| **pancreas** | 5 | 7 | 17 | 4 | 6 | 14 | 22 | 3 | 14 | 25 | 0 |
| **unknown** | 0 | 0 | 0 | 0 | 4 | 0 | 1 | 4 | 0 | 4 | 1 |
| **Σ** | 23 | 22 | 51 | 21 | 48 | 36 | 101 | 28 | 49 | 97 | 19 |

**Supplementary Table 3:** Tumor characteristics II (unkn.: unknown).

| **Origin of the primary tumor** | **Staging (UICC)**  (number of cases) | | | | | **Grading**  (number of cases) | | | |
| --- | --- | --- | --- | --- | --- | --- | --- | --- | --- |
|  | **I** | **II** | **III** | **IV** | **unkn.** | **1** | **2** | **3** | **unkn** |
| **stomach** | 5 | 0 | 1 | 5 | 8 | 5 | 11 | 3 | 0 |
| **duodenum / jejunum** | 4 | 0 | 2 | 8 | 1 | 8 | 6 | 1 | 0 |
| **ileum** | 0 | 0 | 14 | 42 | 3 | 43 | 16 | 0 | 0 |
| **appendix** | 1 | 0 | 1 | 1 | 2 | 3 | 0 | 2 | 0 |
| **colon** | 1 | 0 | 2 | 6 | 0 | 2 | 2 | 5 | 0 |
| **rectum** | 2 | 1 | 1 | 5 | 5 | 2 | 9 | 3 | 0 |
| **pancreas** | 4 | 7 | 2 | 26 | 0 | 14 | 17 | 8 | 0 |
| **unknown** | 0 | 0 | 0 | 4 | 1 | 3 | 0 | 2 | 0 |
| **Σ** | 17 | 8 | 23 | 97 | 20 | 80 | 61 | 24 | 0 |

**Supplementary Table 4:** Immunohistochemical studies on somatostatin receptor expression in gastroenteropancreatic neuroendocrine neoplasms. Func, functionality; Loc, location; G/S, grading/staging; CgA, chromogranin A; Img, SST-based imaging; Surv, Survival; P, pancreatic; GEP, gastroenteropancreatic; GE, gastroenteral; B, bronchopulmonal; n/s, not specified

| **Study** | **Tumor entity** | **Patients (n)** | **Samples (n)** | **SSTs studied** | **Type of antibody** | **Correlation with:** | | | | | | |
| --- | --- | --- | --- | --- | --- | --- | --- | --- | --- | --- | --- | --- |
|  |  |  |  |  |  | **Func** | **Loc** | **G/S** | **Ki-67** | **CgA** | **Img** | **Surv** |
| Kulaksiz et al. 2002 ^(50)^ | GEP | 104 | n/s | 1, 2A, 3, 5 | polyclonal | -- | -- | -- | -- | -- | -- | -- |
| Ono et al. 2007 ^(51)^ | GE, B | 71 | n/s | 1, 2, 3, 5 | polyclonal | -- | -- | -- | -- | -- | -- | -- |
| Volante et al. 2007 ^(33)^ |  | 107 | n/s | 2A, 3, 5 | polyclonal | -- | -- | -- | -- | -- | + | -- |
| Asnacios et al. 2008 ^(52)^ |  | 98 | n/s | all | polyclonal | -- | -- | -- | -- | -- | + | -- |
| Corleto et al. 2009 ^(3)^ | GEP | 33 | 42 | 2A | polyclonal | -- | -- | -- | -- | -- | -- | + |
| Miederer et al. 2009 ^(41)^ | GEP | 18 | n/s | 2A | polyclonal | -- | -- | -- | -- | -- | + | -- |
| Srirjaskanthan et al. 2009 ^(4)^ | GEP | 56 | 56 | 2A, 5 | polyclonal | -- | -- | + | -- | -- | + | -- |
| Zamora et al. 2010 ^(5)^ | GEP | 100 | 100 | all | polyclonal | + | + | + | -- | -- | -- | -- |
| Kaemmerer et al. 2011 ^(37)^ | GEP | 34 | 44 | all | poly-, 2A monoclonal | -- | -- | + | -- | + | + | -- |
| Mizutani et al. 2012 ^(35)^ |  | 32 | n/s | all | polyclonal | -- | + | -- | -- | -- | -- | -- |
| Schmid et al. 2012 ^(53)^ |  | 54 | 67 (TMA) | all | monoclonal | -- | -- | + | + | -- | -- | -- |
| Kaemmerer et al. 2013 ^(54)^ | P | 50 | 66 | 5 | mono- vs. polyclonal | -- | -- | -- | -- | -- | -- | -- |
| Lambertini et al. 2013 ^(55)^ | GEP | 49 | 75 (TMA) | all | monoclonal | -- | -- | -- | -- | -- | -- | -- |
| Okuwaki et al. 2013 ^(6)^ | P | 79 | n/s | 2A | polyclonal | -- | -- | -- | -- | -- | -- | + |
| Kaemmerer et al. 2014 ^(56)^ | GEP | 25 | 40 | all | poly-, 2A monoclonal | -- | -- | -- | -- | -- | + | -- |
| Diakatou et al. 2015 ^(36)^ | GEP, B | 44 | 55 | all | polyclonal | -- | + | -- | -- | -- | + | -- |
| Kaemmerer et al. 2015a ^(7)^ | GEP | 64 | 121 | 1, 2A, 3, 5 | monoclonal | -- | -- | + | + | -- | -- | + |
| Kaemmerer et al. 2015b ^(15)^ | P | 19 | 23 | 2A | monoclonal | -- | -- | -- | -- | -- | + | -- |
| Mehta et al. 2015 ^(8)^ | P | 99 | 99 (TMA) | 2A, 5 | monoclonal | -- | -- | -- | -- | -- | -- | + |
| Qian et al. 2016 ^(9)^ | GEP | 195 | 261 (TMA) | all | mono-, 4 polyclonal | -- | + | -- | + | -- | + | + |
| Song et al. 2016 ^(10)^ | P | 199 | 199 (TMA) | all | polyclonal | + | -- | + | -- | -- | -- | + |
| Wang et al. 2017 ^(12)^ | GEP | 143 | 143 | 2A, 5 | monoclonal | + | + | + | -- | -- | -- | + |
| Brunner et al. 2017 ^(17)^ | GEP, B, other | 279 | n/s (TMA) | 2A | monoclonal | -- | + | -- | -- | -- | + | + |
| Konukiewicz et al. 2017 ^(13)^ | GEP | 47 | 47 | 2A, 5 | polyclonal | -- | -- | + | + | + | -- | -- |
| Herrera-Martinez et al. 2018 ^(57)^ | GEP | 58 | 130 | 1, 2A, 5 | monoclonal | -- | -- | + | -- | -- | -- | -- |
| Mai et al. 2018 (present study) | GEP | 165 | 412 | all | mono-, 4 polyclonal | + | + | + | + | + | + | + |

**References only cited in Supplementary Table 4** (for the other citations see reference list of the main paper)**:**

1. Kulaksiz, H. *et al*. Identification of somatostatin receptor subtypes 1, 2A, 3, and 5 in neuroendocrine tumours with subtype specific antibodies. *Gut*. **50**, 52–60 (2002).
2. Ono, K. *et al*. Somatostatin receptor subtypes in human non-functioning neuroendocrine tumors and effects of somatostatin analogue SOM230 on cell proliferation in cell line NCI-H727. *Anticancer Res*. **27**, 2231–2240 (2007).
3. Asnacios, A. *et al*. Indium-111–pentetreotide scintigraphy and somatostatin receptor subtype 2 expression: new prognostic factors for malignant well-differentiated endocrine tumors. *J. Clin. Oncol*. **28**, 963–970 (2008).
4. Schmid, H. A. *et al*. Monoclonal antibodies against the human somatostatin receptor subtypes 1–5: development and immunohistochemical application in neuroendocrine tumors. *Neuroendocrinol*. **95**, 232–247 (2012).
5. Kaemmerer, D. *et al*. Correlation of monoclonal and polyclonal somatostatin receptor 5 antibodies in pancreatic neuroendocrine tumors. *Int. J. Clin. Exp. Pathol*. **6**, 49–54 (2013).
6. Lambertini, C. *et al*. Evaluation of somatostatin receptor subtype expression in human neuroendocrine tumors using two sets of new monoclonal antibodies. *Regul. Pept*. **187**, 35–41 (2013).
7. Kaemmerer, D. *et al*. Somatostatin receptor immunohistochemistry in neuroendocrine tumors: comparison between manual and automated evaluation. *Int. J. Clin. Exp. Pathol*. **7**, 4971–4980 (2014).
8. Herrera-Martinez, A. D. *et al*. Clinical and functional implication of the components of somatostatin system in gastroenteropancreatic neuroendocrine tumors. *Endocrine*. **59**, 426–437 (2018).

**Supplementary Table 5:** Antibodies used for immunohistochemical stainings (for the stainings of the SST1, SST2A, SST3, SST5 and CXCR4 hybridoma cell culture supernatants were used; commercially available affinity-purified antibodies from Epitomics may be diluted to a greater extent)

| **Antibody** | **Clone** | **Type** | **Epitope** | **Supplier** | **Dilution** |
| --- | --- | --- | --- | --- | --- |
| **SST1** | UMB-7 | rabbit monoclonal | ENLESGGVFRNGTCTSRITTL (residues 377-391) | Epitomics, Burlingame, CA | 1:25 |
| **SST2A** | UMB-1 | rabbit monoclonal | ETQRTLLNGDLQTSI (residues 335-369) | Epitomics, Burlingame, CA | 1:10 |
| **SST3** | UMB-5 | rabbit monoclonal | QLLPQEASTGEKSSTMRISYL (residues 398-418) | Epitomics, Burlingame, CA | 1:20 |
| **SST4** | 4802 | rabbit polyclonal | CQQEALQPEPGRKRIPLTRTTIF (residues 366-388) | Gramsch, Schwabhausen, Germany | 0.1 µg/ml |
| **SST5** | UMB-4 | rabbit monoclonal | QEATPPAHRAAANGLMQTSKL (residues 344-364) | Epitomics, Burlingame, CA | 1:10 |
| **CXCR4** | UMB-2 | rabbit monoclonal | KGKRGGHSSVSTESESSSFHSS (residues 338-359) | Epitomics, Burlingame, CA | 1:2 |
| **Ki-67** | MIB-1 | mouse monoclonal |  | DAKO, Hamburg, Germany | 1:75 |
| **CgA** | LK2H10 | mouse monoclonal |  | BioLogo, Kronshagen, Germany | 1:50 |

**Supplementary Table 6:** Localization of the primary tumor, derivation of the samples investigated and prior treatments of the patients.

| **patient no.** | **gender** | **age at diagnosis** | **localization of primary tumor** | **type of sample(s)** | **treatment** | **prior treatment(s)** |
| --- | --- | --- | --- | --- | --- | --- |
| 1 | male | 43 | appendix | PT | resection | no |
|  |  |  |  | asyn. MTS peritoneum | resection | resection (2 years ago) |
| 2 | female | 52 | appendix | PT | resection | no |
|  |  |  |  | MTS LN | resection |  |
| 3 | male | unkn. | appendix | MTS unkn. | resection | unkn. |
| 4 | male | 66 | appendix | PT | resection | no |
| 5 | male | unkn. | appendix | PT | resection | no |
| 6 | male | 64 | colon sigm. | PT | resection | no |
| 7 | male | 69 | colon desc. | PT | resection | no |
| 8 | male | 68 | ileum | PT | resection | no |
|  |  |  |  | MTS LN | resection |  |
|  |  |  |  | MTS liver | resection |  |
|  |  |  |  | MTS omentum | resection |  |
|  |  |  |  | MTS peritoneum | resection |  |
| 9 | male | 72 | colon asc. | PT | resection | no |
|  |  |  |  | MTS LN | resection |  |
|  |  |  |  | MTS liver | biopsy |  |
| 10 | male | 82 | colon asc. | PT | resection | no |
|  |  |  |  | MTS mesenterium | biopsy |  |
|  |  |  |  | MTS umbilicus | biopsy |  |
| 11 | male | 73 | colon sigm. | PT | resection | resection, 3x PRRT ^177^Lu-DOTA-TATE |
|  |  |  |  | MTS LN | resection |  |
|  |  |  |  | MTS liver | resection |  |
| 12 | female | 62 | colon transv. | MTS liver | biopsy | no |
| 13 | male | 68 | rectum | asyn. MTS adrenal | resection | resections, lanreotide |
| 14 | male | 65 | ileum | PT | resection | octreotide LAR |
|  |  |  |  | MTS LN | resection |  |
| 15 | male | 63 | colon desc. | PT | biopsy | no |
| 16 | male | 53 | duodenum | PT | resection | 4x PRRT ^90^Y-DOTA-TOC |
|  |  |  |  | MTS liver | resection |  |
| 17 | male | 49 | duodenum | asyn. MTS omentum | resection | resection (21 years ago) |
|  |  |  |  | asyn. MTS ileum | resection |  |
|  |  |  |  | asyn. MTS abdominal wall | resection |  |
| 18 | female | 69 | duodenum | asyn. MTS mesenterium | biopsy | 3x PRRT ^177^Lu-DOTA-TATE, octreotide |
| 19 | male | 52 | duodenum | PT | resection | no |
|  |  |  |  | MTS LN | resection |  |
| 20 | female | 67 | duodenum | PT (relapse) | resection | resection (11 years ago), octreotide |
| 21 | female | 43 | duodenum | PT | resection | 1x PRRT ^177^Lu-DOTA-TATE |

| 22 | male | 53 | duodenum | PT | resection | 2x PRRT ^177^Lu-DOTA-TOC |
| --- | --- | --- | --- | --- | --- | --- |
| 23 | female | 60 | duodenum | PT | resection | octreotide |
| 24 | male | 63 | duodenum | PT | resection | partial resection (1 year ago) |
| 25 | female | 47 | duodenum | PT | resection | no |
| 26 | male | 66 | duodenum | PT (relapse) | biopsy | resection (7 years ago) |
| 27 | male | 60 | duodenum | PT | biopsy | no |
| 28 | male | 60 | duodenum | PT | biopsy | no |
| 29 | male | unkn. | duodenum | PT | biopsy | unkn. |
| 30 | female | 53 | colon asc. | PT | resection | no |
|  |  |  |  | MTS LN | resection |  |
| 31 | male | 58 | ileum | PT | resection | 2x PRRT ^177^Lu-DOTA-TATE |
| 32 | female | 57 | ileum | asyn. MTS liver | resection | resection (10 years ago), 4x PRRT ^90^Y-DOTA-TATE, 1x PRRT ^177^Lu-DOTA-TATE |
| 33 | female | 47 | ileum | asyn. MTS liver | biopsy | resection (5 years ago), octreotide LAR, peginterferon alfa-2b, 3x PRRT ^177^Lu-DOTA-TATE |
| 34 | male | 50 | ileum | PT (relapse) | resection | resection (1 year ago), 1x PRRT ^177^Lu-DOTA-TATE |
|  |  |  |  | asyn MTS LN | resection |  |
| 35 | male | 66 | ileum | PT | resection | octreotide LAR, 2x PRRT ^177^Lu-DOTA-TOC, 1x PRRT ^90^Y-DOTA-TOC, 1x PRRT ^177^Lu-DOTA-TATE |
|  |  |  |  | MTS LN | resection |  |
|  |  |  |  | MTS liver | resection |  |
| 36 | male | 59 | ileum | PT | resection | no |
|  |  |  |  | MTS LN | resection |  |
| 37 | female | 61 | ileum | PT | resection | no |
| 38 | male | 58 | ileum | PT | resection | no |
| 39 | male | 51 | ileum | MTS liver | resection | no |
| 40 | female | 49 | ileum | PT | resection | no |
|  |  |  |  | MTS LN | resection |  |
| 41 | female | 62 | ileum | PT | resection | no |
|  |  |  |  | MTS abdominal wall | biopsy |  |
|  |  |  |  | MTS liver | resection |  |
| 42 | male | 56 | ileum | PT | resection | 2x PRRT ^177^Lu-DOTA-TATE |
|  |  |  |  | MTS LN | resection |  |
| 43 | male | 60 | jejunum | MTS ileum | biopsy | 3x PRRT ^177^Lu-DOTA-TATE, 1x PRRT ^177^Lu-DOTA-TOC |
|  |  |  |  | MTS omentum | biopsy |  |
|  |  |  |  | MTS peritoneum | biopsy |  |
|  |  |  |  | MTS liver | biopsy |  |
| 44 | male | 70 | ileum | PT | resection | no |
|  |  |  |  | MTS liver | resection |  |

| 45 | female | 69 | ileum | PT | resection | octreotide LAR,  1x PRRT ^90^Y-DOTA-TATE, 3x PRRT ^177^Lu-DOTA-TATE |
| --- | --- | --- | --- | --- | --- | --- |
|  |  |  |  | MTS liver | resection |  |
|  |  |  |  | MTS mesocolon | resection |  |
| 46 | male | 52 | ileum | asyn. MTS liver | biopsy | resection (22 years ago), octreotide LAR, 5x TACE MTS liver, 3x PRRT ^90^Y-DOTA-TATE, 3x PRRT ^177^Lu-DOTA-TATE |
| 47 | female | 65 | ileum | MTS liver | resection | octreotide, 4x PRRT ^177^Lu-DOTA-TATE |
|  |  |  |  | MTS mesenterium | biopsy |  |
| 48 | female | 81 | ileum | asyn. MTS omentum | biopsy | resection (1 year ago) |
|  |  |  |  | asyn. MTS mesenterium | biopsy |  |
| 49 | female | 49 | ileum | asyn. MTS liver | resection | resection (7 years ago) |
| 50 | female | 65 | ileum | PT (relapse) | resection | resection (2 years ago), 4x PRRT ^90^Y-DOTA-TATE |
| 51 | male | 84 | ileum | PT | resection | octreotide LAR |
|  |  |  |  | MTS liver | resection |  |
| 52 | female | 38 | ileum | MTS liver | resection | no |
|  |  |  |  | MTS ovary | resection |  |
|  |  |  |  | MTS abdominal adipose tissue | biopsy |  |
| 53 | female | 67 | ileum | PT | resection | no |
| 54 | female | 75 | ileum | MTS LN | resection | resection (1 year ago), 1x PRRT ^177^Lu-DOTA-TATE |
| 55 | male | 68 | ileum | PT | resection | 2x PRRT ^177^Lu-DOTA-TOC, 1x PRRT ^90^Y-DOTA-TOC |
|  |  |  |  | MTS liver | resection |  |
|  |  |  |  | MTS abdominal adipose tissue | biopsy |  |
| 56 | male | 68 | ileum | PT | resection | no |
|  |  |  |  | MTS LN | resection |  |
| 57 | male | 58 | ileum | PT | resection | no |
|  |  |  |  | MTS LN | resection |  |
|  |  |  |  | MTS liver | resection |  |
| 58 | male | 58 | ileum | PT | resection | no |
|  |  |  |  | MTS LN | resection |  |
| 59 | male | 50 |  | PT | resection | no |
|  |  |  |  | MTS LN | resection |  |
|  |  |  |  | MTS liver | resection |  |
| 60 | male | 38 | ileum | PT |  | no |
|  |  |  |  | MTS LN |  |  |
| 61 | female | 45 | ileum | asyn. MTS ovary | resection | resection (28 years ago), 7x PRRT ^177^Lu-DOTA-TATE |
| 62 | male | 79 | ileum | PT | resection | no |
|  |  |  |  | MTS LN | resection |  |
| 63 | male | 53 | ileum | PT | resection | 3 cycles cisplatin/etoposide, capecitabine, lanreotide |
|  |  |  |  | MTS LN | resection |  |
|  |  |  |  | MTS liver | resection |  |
| 64 | male | 65 | ileum | PT | resection | no |
|  |  |  |  | MTS LN | resection |  |
|  |  |  |  | MTS liver | resection |  |
| 65 | female | 70 | ileum | PT | resection | octreotide LAR |
|  |  |  |  | MTS LN | resection |  |
|  |  |  |  | MTS liver | resection |  |
| 66 | male | 50 | ileum | PT | resection | no |
| 67 | female | 39 | ileum | asyn. MTS LN | resection | resection (5 years ago), octreotide LAR, 6 cycles BAY-KDR, 2x PRRT ^90^Y-DOTA-TATE, 2x PRRT ^177^Lu-DOTA-TATE |
|  |  |  |  | asyn. MTS ovary | resection |  |
| 68 | female | 64 | ileum | PT | resection | no |
|  |  |  |  | MTS LN | resection |  |
| 69 | male | 51 | ileum | PT | resection | no |
|  |  |  |  | MTS LN | resection |  |
| 70 | female | 62 | ileum | PT | resection | 4x TACE MTS liver, octreotide LAR |
| 71 | female | 77 | ileum | PT | resection | no |
|  |  |  |  | MTS LN | resection |  |
| 72 | female | 56 | ileum | PT | resection | octreotide LAR, 2x PRRT ^90^Y-DOTA-TOC, 2x PRRT ^177^Lu-DOTA-TATE |
| 73 | male | 70 | ileum | PT | resection | 2x PRRT ^177^Lu-DOTA-TOC |
|  |  |  |  | MTS LN | resection |  |
| 74 | male | 69 | ileum | PT | resection | no |
|  |  |  |  | MTS liver | resection |  |
| 75 | female | 53 | ileum | PT | resection | octreotide LAR, telotristate-etiprate (TELESTAR study) |
|  |  |  |  | MTS LN | resection |  |
|  |  |  |  | MTS liver | resection |  |
| 76 | male | 59 | ileum | PT | resection | no |
|  |  |  |  | MTS LN | resection |  |
| 77 | female | 64 | ileum | PT | resection | resection MTS liver |
|  |  |  |  | MTS LN | resection |  |
|  |  |  |  | MTS liver | biopsy |  |
| 78 | female | 80 | ileum | PT | resection | octreotide LAR, 3x PRRT ^177^Lu-DOTA-TOC |
|  |  |  |  | MTS LN | resection |  |
|  |  |  |  | MTS liver | biopsy |  |
| 79 | female | 77 | ileum | PT | resection | no |
|  |  |  |  | MTS LN | resection |  |
| 80 | male | 54 | ileum | PT | resection | no |
| 81 | male | 70 | ileum | PT | resection | no |
| 82 | female | 74 | ileum | PT | resection | no |
| 83 | female | 50 | ileum | PT | resection | octreotide LAR, 1x PRRT ^90^Y-DOTA-TATE, 2x PRRT ^177^Lu-DOTA-TATE |
|  |  |  |  | MTS LN | resection |  |
| 84 | male | 53 | ileum | PT | resection | no |
| 85 | male | 58 | ileum | PT | resection | no |
| 86 | female | 56 | ileum | PT | resection | 2x PRRT ^177^Lu-DOTA-TATE, 2x PRRT ^90^Y-DOTA-TATE |
|  |  |  |  | MTS mesenterium | biopsy |  |
|  |  |  |  | MTS peritoneum | biopsy |  |
|  |  |  |  | MTS liver | biopsy |  |
|  |  |  |  | MTS ovary | resection |  |
| 87 | male | 45 | stomach | PT | resection | no |
| 88 | female | 49 | stomach | asyn. MTS liver | resection | resection (14 years ago), 6x chemoembolization MTS liver, 5x PRRT ^90^Y-DOTA-TATE |
|  |  |  |  | asyn. MTS gallbladder | resection |  |
| 89 | male | 50 | stomach | MTS lig. hepatoduodenale | resection | no |
| 90 | female | 58 | stomach | PT | biopsy | no |
| 91 | male | 84 | stomach | PT | resection | unkn. |
| 92 | female | 38 | stomach | PT | resection | unkn. |
| 93 | female | 57 | stomach | PT | resection | no |
| 94 | female | 64 | stomach | PT | biopsy | no |
| 95 | female | 57 | stomach | PT | resection | no |
| 96 | female | 65 | pancreas | PT | biopsy | streptozotocin/5-fluorouracil, 4x PRRT ^177^Lu-DOTA-TATE |
| 97 | female | 82 | stomach | PT | biopsy | no |
| 98 | male | 74 | ileum | asyn. MTS liver | biopsy | resection (4 years ago), octreotide |
| 99 | male | 78 | stomach | PT | biopsy | no |
| 100 | female | 46 | pancreas | asyn. MTS stomach | biopsy | resection (3 years ago), 10x TACE MTS liver, everolimus (RADIANT-3 study) |
|  |  |  |  | asyn. MTS omentum majus | resection |  |
|  |  |  |  | asyn. MTS mesocolon | resection |  |
|  |  |  |  | asyn. MTS abdomen | resection |  |
| 101 | female | 21 | stomach | PT | biopsy | 1 cycle cisplatin/ etoposide |
| 102 | male | 67 | stomach | PT | biopsy | no |
| 103 | female | 66 | stomach | PT | resection | unkn. |
| 104 | male | 41 | stomach | PT | biopsy | no |
| 105 | female | 71 | stomach | PT (relapse) | biopsy | resection, additional follicular lymphoma (chlorambucil, 6 cycles CHOP, rituximab) |
| 106 | female | 62 | stomach | PT | biopsy | no |
| 107 | female | 64 | stomach | PT | biopsy | no |
| 108 | female | 52 | pancreas | asyn. MTS liver | biopsy | octreotide LAR, 13 cycles dacarbazine, 3x PRRT ^177^Lu-DOTA-TATE, 1x PRRT ^90^Y-DOTA-TATE, 2x TACE MTS liver |
| 109 | female | 59 | pancreas | PT | resection | no |
|  |  |  |  | MTS LN | resection |  |
| 110 | male | 44 | stomach | asyn. MTS liver | biopsy | resection (5 years ago), 2 cycles cisplatin/etoposide, octreotide LAR, interferon, 2x PRRT ^90^Y-DOTA-TATE, 1x PRRT ^177^Lu-DOTA-TATE |
| 111 | female | 32 | pancreas | PT | resection | 2x PRRT ^90^Y-DOTA-TATE |
|  |  |  |  | asyn. MTS LN | biopsy | PRRT and resection (6 years ago) |
| 112 | male | 51 | pancreas | PT | resection | no |
|  |  |  |  | MTS LN (mult.) | resection |  |
| 113 | female | 43 | pancreas | MTS liver | resection | 3x PRRT ^90^Y-DOTA-TATE |
| 114 | female | 59 | pancreas | PT | resection | no |
|  |  |  |  | PT (relapse) | resection | resection (5 years ago), interferon, 4x PRRT ^90^Y-DOTA-TATE |
| 115 | male | 53 | pancreas | PT | resection | no |
| 116 | male | 71 | pancreas | PT | resection | no |
| 117 | male | 75 | pancreas | asyn. MTS stomach | biopsy | resection (2 years ago) |
| 118 | female | 73 | pancreas | PT | resection | no |
| 119 | male | 50 | pancreas | PT | resection | no |
|  |  |  |  | asyn. MTS liver | resection | resection (9 years ago), octreotide LAR, interferon, 5x TACE MTS liver, resection MTS thyroid, 3x PRRT ^90^Y-DOTA-TATE |
| 120 | male | 55 | pancreas | PT | resection | 4x PRRT ^177^Lu-DOTA-TATE |
|  |  |  |  | asyn. MTS liver | resection | PRRT and resection (2 years ago) |
| 121 | female | 60 | pancreas | PT | resection | 2 cycles fluorouracil/ streptozotocin |
|  |  |  |  | MTS liver | biopsy |  |
| 122 | female | 37 | pancreas | PT | resection | no |
|  |  |  |  | MTS liver | resection |  |
|  |  |  |  | asyn. MTS LN (mult.) | biopsy | resection (11 years ago), octreotide LAR, 4x PRRT ^90^Y-DOTA-TATE |
|  |  |  |  | asyn. MTS suet | biopsy |  |
| 123 | female | 48 | pancreas | PT | resection | no |
|  |  |  |  | MTS LN | resection |  |
|  |  |  |  | MTS liver | resection |  |
| 124 | female | 43 | pancreas | PT | resection | 3x PRRT ^90^Y-DOTA-TATE |
|  |  |  |  | MTS liver | resection |  |
| 125 | female | 33 | pancreas | PT | resection | no |
|  |  |  |  | asyn. MTS liver | biopsy | resection (2 years ago), octreotide LAR |
| 126 | male | 40 | pancreas | PT | resection | 7 cycles temozolomide/ capecitabine, 1x PRRT ^177^Lu-DOTA-TATE, 2x PRRT ^90^Y-DOTA-TOC |
|  |  |  |  | MTS liver | resection |  |
| 127 | male | 63 | pancreas | PT | resection | no |
| 128 | male | 49 | pancreas | PT | resection | 6 cycles carboplatin/ etoposide, lanreotide, everolimus, 4x PRRT ^90^Y-DOTA-TATE |
|  |  |  |  | MTS LN | resection |  |
|  |  |  |  | MTS liver | biopsy |  |
| 129 | male | 53 | pancreas | PT (relapse) | resection | resection (10 years ago), 1x PRRT ^177^Lu-DOTA-TOC, 1x PRRT ^90^Y-DOTA-TOC, 1x PRRT ^177^Lu-HA-DOTA-TATE |
|  |  |  |  | asyn. MTS liver | resection |  |
| 130 | female | 75 | pancreas | PT | resection | no |
| 131 | male | 70 | pancreas | PT | resection | no |
|  |  |  |  | MTS liver | biopsy |  |
|  |  |  |  | asyn. MTS liver | resection | resection (1 year ago) |
| 132 | male | 49 | pancreas | PT | resection | 2x PRRT ^99^Y-DOTA-TATE, 1x PRRT ^177^Lu-DOTA-TOC + capecitabine |
|  |  |  |  | MTS liver | biopsy |  |
| 133 | female | 65 | pancreas | PT | resection | no |
| 134 | female | 40 | pancreas | asyn. MTS liver | resection | resection (3 years ago), 2x PRRT ^90^Y-DOTA-TATE, 1x PRRT ^177^Lu-DOTA-TATE, 1x PRRT ^177^Lu-DOTA-TOC, 1x PRRT ^90^Y-DOTA-TOC |
| 135 | male | 58 | pancreas | PT | resection | no |
| 136 | male | 72 | pancreas | PT | resection | no |
| 137 | male | 58 | pancreas | PT | resection | 21 cycles FOLFOX, 1x PRRT ^90^Y-DOTA-TATE, 1x PRRT ^177^Lu-DOTA-TOC, 1x PRRT ^90^Y-DOTA-TOC |
|  |  |  |  | MTS liver | biopsy |  |
| 138 | male | 53 | ileum | asyn. MTS peritoneum | biopsy | resection (3 years ago), capecitabine, octreotide LAR, 3x PRRT ^177^Lu-DOTA-TOC |
| 139 | male | 60 | pancreas | PT | resection | no |
| 140 | male | 72 | pancreas | PT | resection | no |
| 141 | male | 12 | CUP | MTS liver | resection | no |
| 142 | male | 15 | pancreas | PT | resection | no |
| 143 | male | 56 | pancreas | PT | resection | mult. resections MTS LN/liver, octreotide LAR, 1x PRRT ^177^Lu-DOTA-TATE, 2x PRRT ^177^Lu-DOTA-TOC |
| 144 | male | 80 | pancreas | PT | resection | no |
| 145 | male | 45 | pancreas | PT | resection | no |
| 146 | female | 41 | pancreas | PT | resection | no |
| 147 | male | 52 | rectum | PT | resection | no |
|  |  |  |  | MTS LN | resection |  |
| 148 | female | 70 | rectum | PT | resection | no |
| 149 | male | 55 | rectum | PT | resection | no |
|  |  |  |  | MTS liver | resection |  |
|  |  |  |  | MTS thyroid | resection |  |
| 150 | female | 67 | rectum | PT | resection | no |
|  |  |  |  | MTS LN | resection |  |
|  |  |  |  | MTS liver | resection |  |
| 151 | male | 28 | rectum | PT | resection | no |
| 152 | male | 56 | rectum | PT | resection | 2x PRRT ^90^Y-DOTA-TOC, 2x PRRT ^177^Lu-DOTA-TOC |
| 153 | female | 61 | rectum | PT | resection | no |
| 154 | male | 75 | rectum | PT | resection | unkn. |
| 155 | male | 48 | rectum | PT | resection | unkn. |
| 156 | female | 85 | rectum | PT | resection | unkn. |
| 157 | male | 82 | rectum | PT | resection | unkn. |
| 158 | female | 69 | rectum | MTS liver | biopsy | pasireotide/ everolimus (COOPERATE-1-trial) |
| 159 | male | 55 | rectum | MTS liver | biopsy | pasireotide/ everolimus (COOPERATE-1-trial) |
| 160 | male | 73 | CUP | MTS retroperitoneum | resection | no |
|  |  |  |  | MTS skin | resection |  |
| 161 | female | 55 | unkn. (gut multifocal) | asyn. MTS liver | resection | resection (5 years ago), octreotide LAR, 2x PRRT ^90^Y-DOTA-TATE |
| 162 | male | 59 | CUP | MTS peritoneum | biopsy | resection MTS liver (2 years ago), octreotide LAR |
| 163 | male | 78 | unkn. | unkn. | resection | unkn. |
| 164 | female | 58 | pancreas | PT | resection | no |
|  |  |  |  | MTS liver | resection |  |

| 165 | male | 75 | colon transv. | PT | resection | no |
| --- | --- | --- | --- | --- | --- | --- |
|  |  |  |  | MTS LN | resection |  |
|  |  |  |  | asyn. MTS LN |  | resection (1 year ago) |

asyn.: asynchronous, CUP: cancer of unknown primary, DOTA: 1,4,7,10-tetraazacyclododecane-1,4,7,10-tetra acetic acid, DOTA-TATE: DOTA-(Tyr^3^)-octreotate, DOTA-TOC: (DOTA^0^-Phe^1^-Tyr^3^)octreotide, FOLFOX: folinic acid, 5-fluorouracil, oxaliplatin, LAR: long acting release, LN: lymph node(s), MTS: metastasis/es, mult.: multiple, PRRT: peptide receptor radionuclide therapy, PT: primary tumor(s), unkn.: unknown, TACE: transarterial chemoembolization
